# Multimodal Neural Response and Effect Assessment During a BCI-Based Neurofeedback Training After Stroke

**DOI:** 10.3389/fnins.2022.884420

**Published:** 2022-06-17

**Authors:** Zhongpeng Wang, Cong Cao, Long Chen, Bin Gu, Shuang Liu, Minpeng Xu, Feng He, Dong Ming

**Affiliations:** ^1^Academy of Medical Engineering and Translational Medicine, Tianjin University, Tianjin, China; ^2^Department of Biomedical Engineering, College of Precision Instruments and Optoelectronics Engineering, Tianjin University, Tianjin, China; ^3^Tianjin International Joint Research Center for Neural Engineering, Tianjin, China

**Keywords:** brain computer interface, neurofeedback training, electroencephalography, functional electrical stimulation, functional near-infrared spectroscopy, stroke

## Abstract

Stroke caused by cerebral infarction or hemorrhage can lead to motor dysfunction. The recovery of motor function is vital for patients with stroke in daily activities. Traditional rehabilitation of stroke generally depends on physical practice under passive affected limbs movement. Motor imagery-based brain computer interface (MI-BCI) combined with functional electrical stimulation (FES) is a potential active neural rehabilitation technology for patients with stroke recently, which complements traditional passive rehabilitation methods. As the predecessor of BCI technology, neurofeedback training (NFT) is a psychological process that feeds back neural activities online to users for self-regulation. In this work, BCI-based NFT were proposed to promote the active repair and reconstruction of the whole nerve conduction pathway and motor function. We designed and implemented a multimodal, training type motor NFT system (BCI-NFT-FES) by integrating the visual, auditory, and tactile multisensory pathway feedback mode and using the joint detection of electroencephalography (EEG) and functional near-infrared spectroscopy (fNIRS). The results indicated that after 4 weeks of training, the clinical scale score, event-related desynchronization (ERD) of EEG patterns, and cerebral oxygen response of patients with stroke were enhanced obviously. This study preliminarily verified the clinical effectiveness of the long-term NFT system and the prospect of motor function rehabilitation.

## Introduction

Stroke is the fifth leading cause of death in the world and one of the major causes of acquired disability in adults (Benjamin et al., 2019). Stroke has devastating effects on many survivors, including severe motor and sensory disorders, which hinder their daily activities (Kim et al., 2020). However, the traditional rehabilitation training methods are mostly single passive treatment, and the curative effect is not ideal.

In recent years, brain-computer interface (BCI) (Wolpaw et al., 2000; Wolpaw and Wolpaw, 2012) as a new human-computer interaction technology can provide users with a direct way of communication between the brain and the external environment. Motor imagery-based BCI (MI-BCI) can induce the improvement of motor learning ability and plasticity rehabilitation according to the principle that motor imagery can produce the activation characteristics of sensorimotor cortex similar to motor execution (Várkuti et al., 2013), which is widely used in the rehabilitation of patients with stroke. Neurofeedback training (NFT) refers to the user's control of brain activation and regulation of brain consciousness (Ang and Guan, 2013). Its significance is to observe and understand the characteristic mechanism of the nervous system in order to manipulate some behavior-related neural processes, especially for patients with nerve injury (Sitaram et al., 2017). Therefore, the combination of MI-BCI and NFT is a very potential method for the rehabilitation of patients with stroke.

Many studies have shown that MI-BCI treatment can trigger long-term neurological changes in the upper limbs of patients with stroke and improve their motor function (Cervera et al., 2018; Lyukmanov et al., 2018; Ramos-Murguialday et al., 2019). In addition, MI combined with external mechanical assist devices can make the affected limb perform corresponding actions, which can produce corresponding event-related desynchronization/synchronization (ERD/ERS) and enhance the activity pattern of the brain (Tam et al., 2011; Yao et al., 2019; Aljalal et al., 2020). Jang et al. (2016) investigated the effects of BCI combined with functional electrical stimulation (FES) training on shoulder subluxation of patients with stroke and demonstrated that BCI-FES training can effectively improve shoulder subluxation in patients with stroke by promoting exercise recovery. Biasiucci et al. (2018) divided patients with stroke into two groups, namely, BCI-FES group and the sham-FES group. Patients who tried to move their affected hand in BCI-FES group were given a positive feedback and random FES stimulation in sham-FES group; the result showed only BCI-FES groups improved function and increased functional connectivity between affected hemispheric motion regions.

Electroencephalography (EEG) is the most common measurement and evaluation tool in BCI. To further evaluate the activation status and physiological changes of the brain, many studies have analyzed physiological signals from functional near-infrared spectroscopy (fNIRS) (Kaiser et al., 2014; Chowdhury et al., 2019). Simis et al. (2018) demonstrated fNIRS is suitable for measuring the brain activity of patients with spinal cord injury during robotic walking. Leamy et al. (2011) combined EEG and fNIRS signal features to improve the classification accuracy of MI. Some studies have confirmed the potential of multimodal EEG/fNIRS technology in monitoring and predicting motor recovery after stroke (Li et al., 2017, 2020).

In our previous study, we collected EEG and fNIRS of healthy subjects under NFT to study the mechanism of neural response. The results showed that MI-BCI combined with FES could induce stronger brain electrophysiological and hemodynamic response (Wang et al., 2019). In this study, we designed a BCI-NFT-FES stroke rehabilitation application system with EEG, fNIRS joint acquisition and visual, auditory, and tactile feedback and carried out a long-term clinical trial on typical patients with stroke to realize the rehabilitation training induced by subjective motor intention. Through the analysis of clinical scale, brain electrophysiology, and brain blood oxygen level, the feasibility and effectiveness of long-term multisensory NFT are verified.

## Materials and Methods

### Participants

We recruited 7 patients with stroke from the rehabilitation department of Tianjin People's Hospital (3 males and 4 females, age range: 40–65 years old, all in stable recovery period). Seven patients were randomly divided into experimental group (2 males and 2 females) and control group (1 male and 2 females) according to gender, age, and patients' conditions. We also recruited 7 healthy subjects (3 males, right-handed, age range: 27–45 years) from Tianjin University as the healthy control group. All subjects were double-blinded. The procedure of the experiment was clearly explained to each subject before data recording. This study was approved by the ethical committee of Tianjin University and Tianjin People's Hospital. Consent form was obtained from each subject prior to the experiment. The information of patients with stroke is shown in Table 1.

**Table 1:** Basic information of stroke patients participating in this study.

	**Gender**	**Age**	**Stroke duration**	**Site of lesion**	**Side of hemiparesis**	**MSA/MBI**	**Group**
*S1*	M	40	5 months	BG	L	2/50	EG
*S2*	W	64	3 months	CO, BG	R	1–2/30	EG
*S3*	W	56	4 months	BG	R	2–/40	EG
*S4*	M	40	6 months	BG, CO, Th	L	2–/60	EG
*S5*	W	56	5 months	BG	R	2/60	CG
*S6*	M	65	3 months	BG, CO	L	2–/30	CG
*S7*	W	55	3 months	BG	R	2/40	CG

*BG, basal ganglia; CO, centrum ovale; Th, thalamus; MSA, Muscle strength assessment; MBI, Modified Barthel Index; EG, Experience group; CG, Control group*.

### Design of Experimental Paradigm

**Figure 1:**
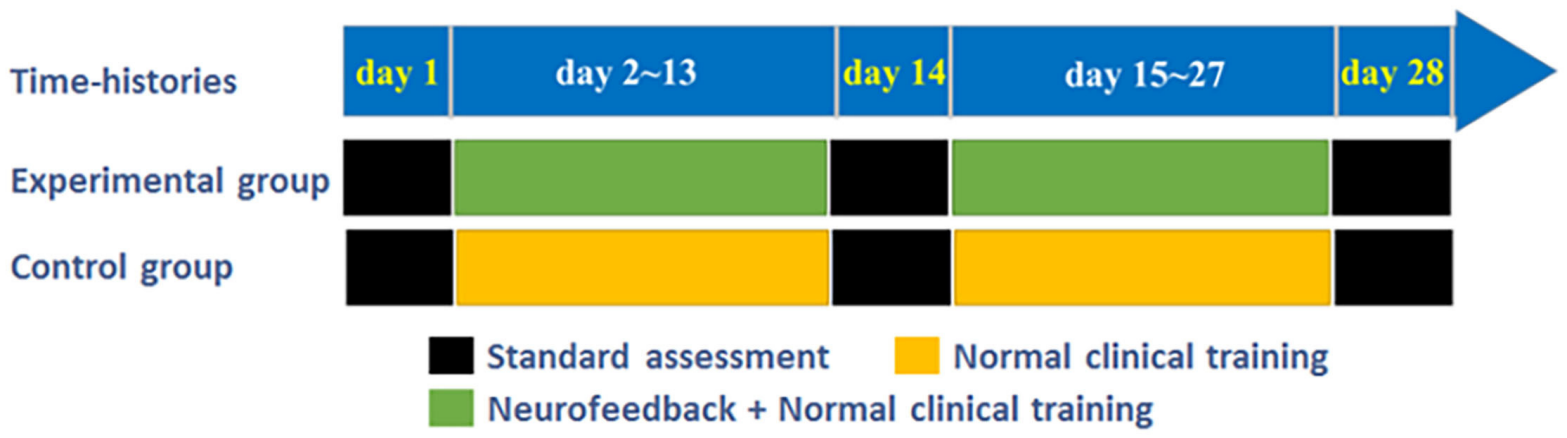
Schematic diagram of long term NFT experiment and control experiment arrangement.

The experimental training task flow is shown in Figure 1. Each patient needs to participate in and complete the exercise training task of the affected limb for 4 weeks. The experimental group patients participate in three standard assessment sessions. Before, during, and after the experimental training process, the Graz motor imagery-based BCI (Graz MI-BCI) paradigm (Mueller-Putz et al., 2014) was utilized. Considering that the change of cerebral blood flow is a slow process, we set the preparation time before each trial to 30 s to ensure that the blood oxygen concentration returns to the baseline state. The NFT session was carried out three times a week (one time every other day, 12 times in total). During this period, they continued to receive routine clinical exercise rehabilitation training. The healthy control group only participated in the Graz MI-BCI and NFT. Compared with the patients in the experimental group, the patients in the control group were also given three Graz MI-BCI but only received the same routine clinical exercise rehabilitation training as the patients in the experimental group during the training period.

**Figure 2:**
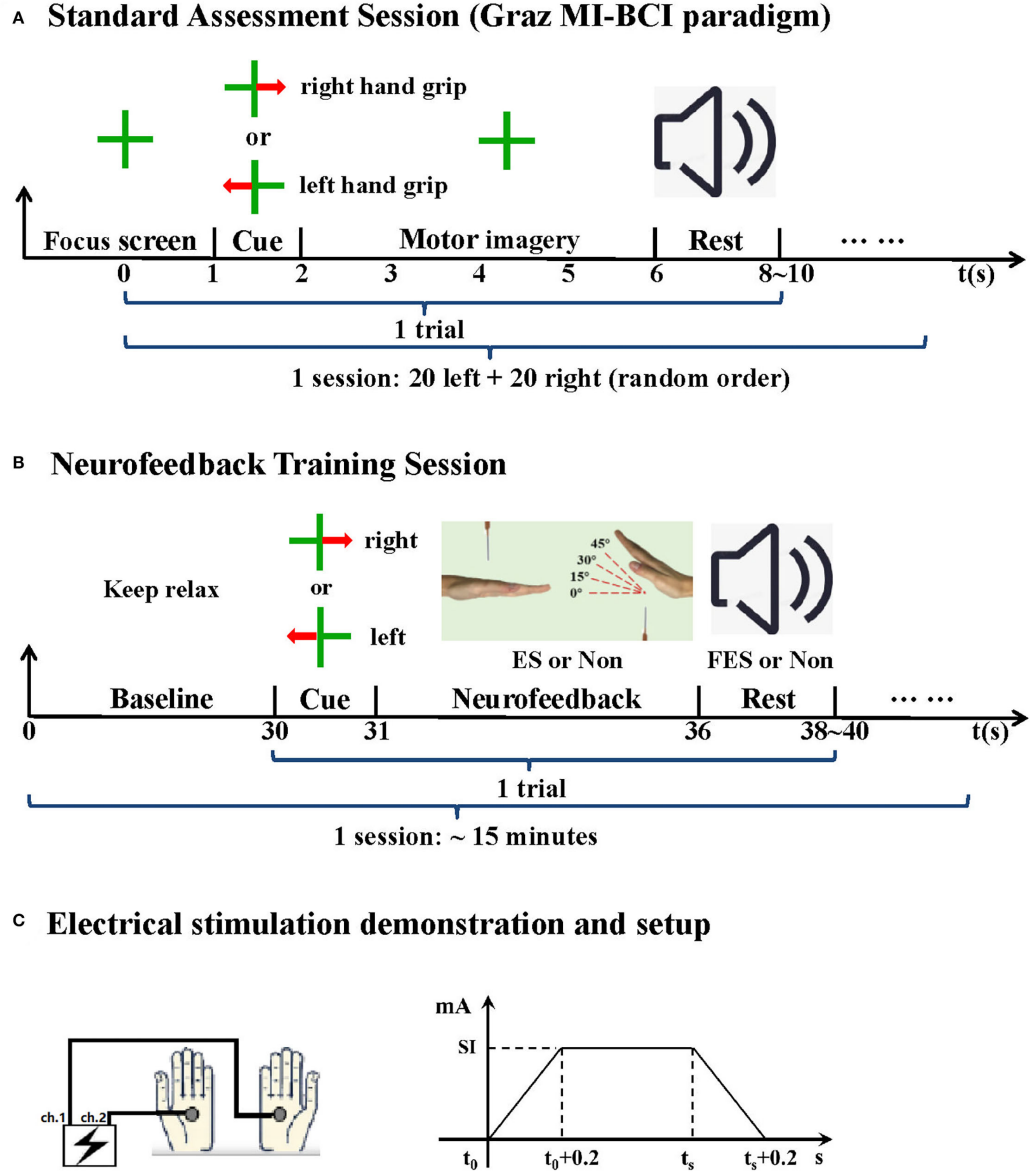
Experimental paradigm. **(A)** Standard assessment session. **(B)** NFT session. **(C)** Electrical stimulation, SI indicates stimulation intensity individually determined, ts indicates the end time of current NFT trial or the time when the training feature parameter lower than the threshold again.

The specific system integration scheme is designed as follows: Graz MI-BCI paradigm in the standard assessment session is shown in Figure 2A, which is divided into two sessions (i.e., one calibration session and one online feedback session, 40 trials each). The offline model is established by using the data in calibration session. The offline modeling method follows the classical SVM + CSP method used in our previous research (Wang et al., 2019). For each trial, the first stage is the preparation stage. A green cross appears on the LCD screen display for 1 s, and then a red arrow corresponding to the direction of the affected limb appears on the screen, indicating the MI of the corresponding upper limb (for the healthy subjects, the imaginary right hand task mode was used). After 1 s, it turns into a green cross again, indicating that the subjects need to perform the prompted motor imagery task, and the task lasts for 4 s. The subjects are required to imagine the wrist extension of the corresponding limb and then enter the rest time of 6–8 s. In online feedback session, the decision results of the imagination task will be fed back through voice.

The NFT session is shown in Figure 2B, each lasting for about 15 min. Each trial mainly contains a penalty stage and a reward stage. First is the 30 s baseline period, during which the data were used to calculate the threshold of NFT initial feature parameter. Then, there is a one second arrow prompt, which tells the subjects to perform left-hand or right-hand MI. Next is the penalty stage (MI stage); there are four moving angles of virtual hand in the screen, namely, 0°, 15°, 30°, and 45° for the real-time feedback training feature parameter level. If the training parameter was below the threshold (Goal_1, up to 15°), the virtual hands changed their angles to get away from the needles. The angles of the virtual hands increased whenever the feedback parameter stayed below the threshold for more than 2 s (Goal_2, up to 30°) and 3 s (Goal_3, up to 45°). Thus, the ultimate goal for the subjects was to keep away from the needles as far as possible. On the contrary, if the training parameter was above the threshold [the needles touched the hands (t_0_), 0°], an electrical stimulation would be applied to left- and right-hand palms (reminding the subject to have a self-regulation) until the needle moved away from the hand or this trial ended (t_s_). The stimulation parameters are shown in Figure 2C. Here, electrical stimulation is used as real-time tactile feedback to induce subjects to adopt more effective dynamic motor imagination strategies. Subjects can appropriately adjust the imagination strategies to achieve the goal of neurofeedback as much as possible. Theoretically, a more optimized dynamic motor imagination strategy can achieve stronger sensory motor cortex activation characteristics and higher system classification performance (Mehler et al., 2018). The last is the reward stage, the subjects only need to keep resting, the system makes a decision on the penalty stage data according to the previously established offline model, and the decision result will be notified by voice. If the decision result is correct, the corresponding limb FES will be given to induce wrist extension.

Functional electrical stimulation equipment is a self-made equipment, which has passed the China Food and Drug Administration (CFDA) registration test. The stimulation frequency is 30 Hz, and the stimulation waveform is 300 μs. The intensity depends on the specific situation of the subjects. Stimulation-related muscles include palmaris longus, flexor carpi ulnaris, and flexor digitorum superficialis. In the stage of nerve feedback punishment, the electrodes were placed on the palms of both sides of the subjects. In the stage of nerve feedback reward, according to the distribution structure of human muscles, the electrodes were placed on the inside of the forearm (the distance between the electrodes is about 10 cm) to promote the corresponding action of the target limb. In addition, all the experimental paradigm interfaces of this study were compiled with the special toolbox of MATLAB.

### Calculation of NFT Feature Parameter

**Figure 3:**
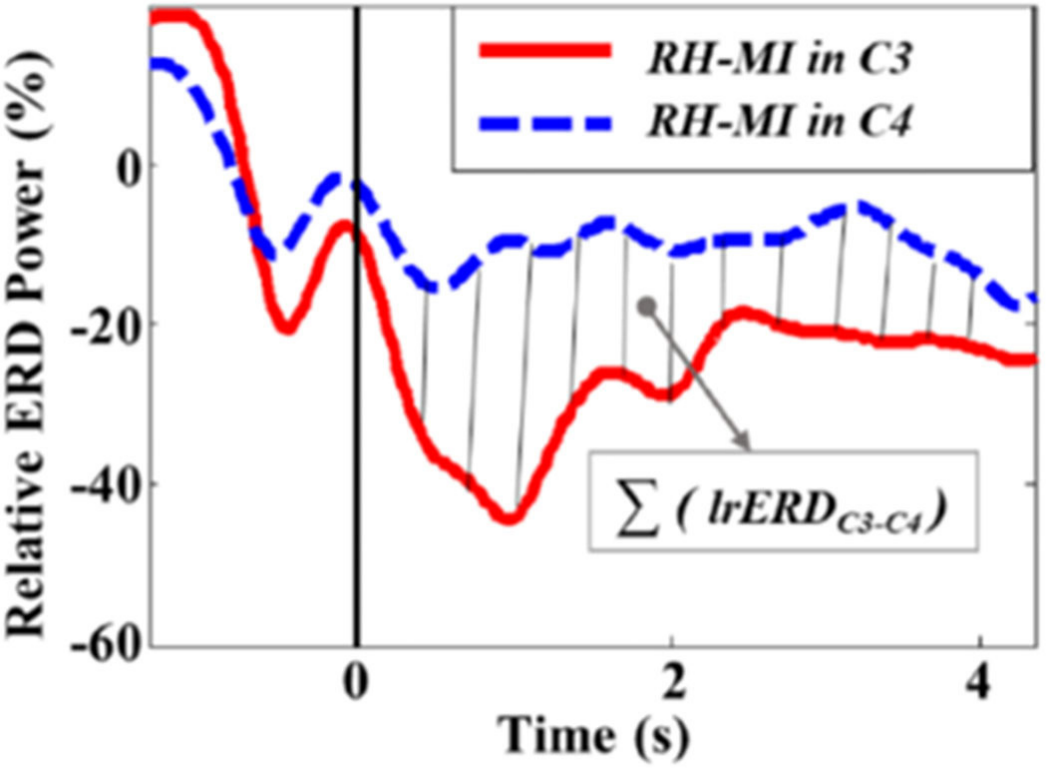
lrERD calculation diagram of RH-MI training.

For left-hand MI (LH-MI) or right-hand MI (RH-MI) training, we proposed and adopted the lateralized relative ERD (lrERD) as the feature parameter of NFT. In EEG-based BCI research, C3, C4, and Cz are demonstrated to be optimal for recognizing MI states (Wang et al., 2007). C3 and C4 are the classic channels for the activation of brain areas in motor-related tasks and also the classic channels for the analysis of ERD/ERS pattern, which are closely related to the activation state and level of human sensory motor cortex (Nakayashiki et al., 2014; Meng and He, 2019). Therefore, first, the average lrERD power (including two typical frequency bands of C3 and C4 channels, alpha: 8–13 Hz and beta: 14–29 Hz) of the baseline resting period (30 s) was selected as the initial threshold, and then the real-time lrERD power between C3 and C4 channels is calculated in the NFT penalty stage, taking RH-MI training as an example, as shown in Figure 3.

The calculation formula of RH-MI training parameters is as follows:


(1) $$\begin{array}{l} lrERD_{C3-C4}=RP_{ERD,C3}-RP_{ERD,C4} \end{array}$$

The calculation formula of LH-MI training parameters is as follows:


(2) $$\begin{array}{l} lrERD_{C4-C3}=RP_{ERD,C4}-RP_{ERD,C3} \end{array}$$

The relative ERD power (*RP*_*ERD*_) is a relative power value, expressed in percentage. *RP*_*ERD*_ is calculated by the power of *n* trials across task or relax period (*Pn*) (Nakayashiki et al., 2014), which is defined as follows:


(3) $$\begin{array}{l} P_{relax}=\frac{1}{T_{relax}}\underset{n\in T_{relax}}{\sum }P_{n} \end{array}$$

(4) $$\begin{array}{l} P_{task}=\frac{1}{T_{task}}\underset{n\in T_{task}}{\sum }P_{n} \end{array}$$

(5) $$\begin{array}{l} RP_{ERD}=\frac{P_{task}-P_{relax}}{P_{relax}}\times 100 \end{array}$$

where *P*_*relax*_ and *P*_*task*_ are the average power spectra during the rest period (*T*_*relax*_) and the task period (*T*_*task*_). Therefore, it is necessary to calculate the time-frequency characteristic power related to events and introduce the event-related spectral perturbation (ERSP) method to calculate the ERD/ERS characteristic power of different tasks and frequency bands, so as to calculate *P*_*n*_, *P*_*relax*_, and *P*_*task*_. Its definition formula is as follows:


(6) $$\begin{array}{l} ERSP\left(f,t\right)=\frac{1}{n}\overset{n}{\underset{k=1}{\sum }}\left(F_{k}\left(f,t\right)\right) \end{array}$$

where *F*_*k*_ (*f, t*) represents the spectral estimation at frequency *f* and time *t* for the *k*th trial. The ERSP was computed through short-time Fourier transform (STFT) with a 256-point Hanning-tapered window from EEGLAB (Nakayashiki et al., 2014; Yi et al., 2017).

### Multimodal Neural Signal Acquisition

**Figure 4:**
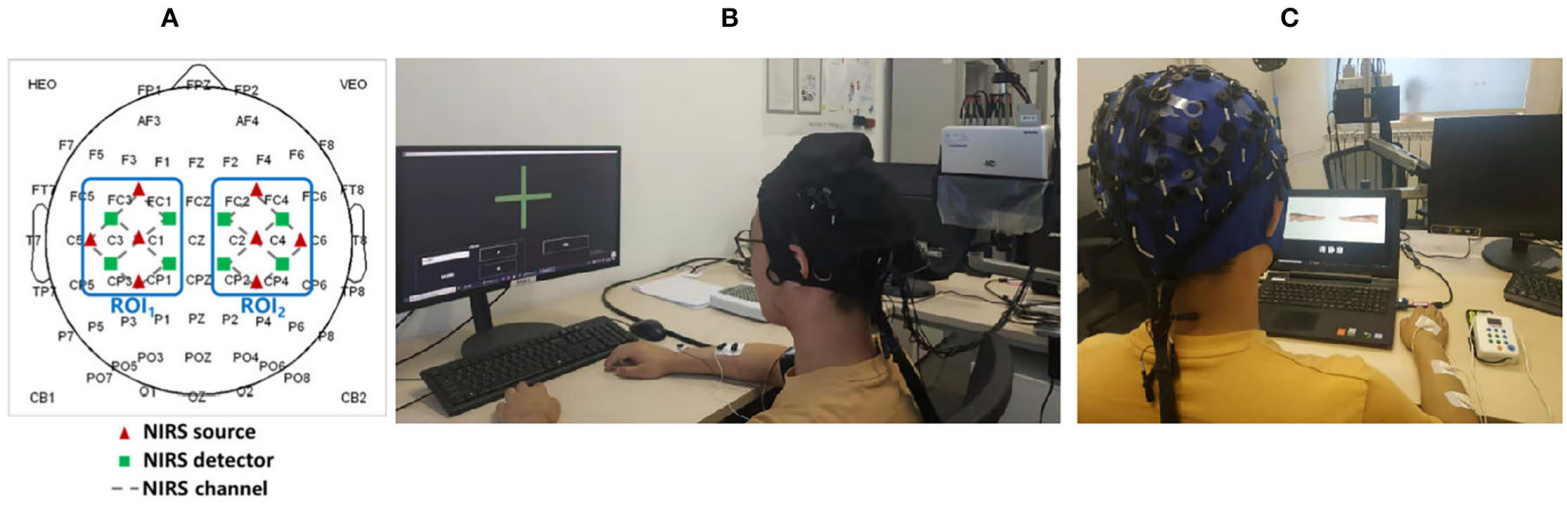
**(A)** Signal acquisition sensor configuration **(B)** Standard assessment session **(C)** Multimodal NFT session.

The subjects wore EEG-fNIRS-combined acquisition head cap (EEG electrophysiology and fNIRS cerebral blood oxygen signals can be collected at the same time) on the head for the experimental session. The specific signal acquisition sensor configuration is shown in Figure 4A. EEG signals were acquired by a 64-channel SynAmps2 system (Quik Cap, Neuroscan, USA) with standard Ag/AgCl electrodes placed on the scalp according to the international 10–20 system. The reference electrode was placed on the nose and the ground electrode was placed on the forehead. The impedance for all electrodes was kept below 10 kΩ. The fNIRS signals acquisition: the distance between the source probe and the detector probe of fNIRS channel is 3 cm, and the excitation mode of the light source is a standard three wavelength laser source (i.e., 780, 808, and 850 nm). The two signal acquisition channels cover the left- and right-hand movement-related sensory motor areas (ROI1 and ROI2). The standard assessment and NFT scenes are shown in Figures 4B,C.

### Clinical Evaluation Methods

According to the three standard assessment sessions of patients with stroke in this study, the first step was to evaluate the classic clinical scale, i.e., (1) muscle strength assessment and (2) modified Barthel index. The former is based on the antigravity motion amplitude and antiresistance motion amplitude, and the muscle strength is graded from 0 to 100% (grades 0–5, the higher the grade, the closer the muscle strength assessment is to normal). If the measured muscle strength is slightly stronger than a certain level, add “+” in the upper right corner, add “–” in the upper right corner if it is slightly worse. The latter is used to assess activities of daily living, using the 100-point system (0–20 = very serious dysfunction, 25–45 = serious dysfunction, 50–70 = moderate functional defect, 75–95 = mild functional defect, 100 = ADL self-care).

### EEG Data Processing and Analysis

In this study, the special EEG processing toolbox EEGLAB of the MATLAB software was used to preprocess the original EEG signal. The steps are as follows: (1) data downsampling to 200 Hz; (2) adopting third-order Butterworth band pass filter (5–35 Hz); and (3) defining the appearance time of the imagination prompt interface as zero time, taking the event code as the signal sign, and intercepting the data from 2 s before zero time to 5 s after zero time.

### fNIRS Data Processing and Analysis

The preprocessing of cerebral blood oxygen signal uses a professional software (Homer2) to process and analyze the data. The specific preprocess includes the following steps. (1) Manual artifact removal of original data. (2) The original light intensity signal is converted to optical density (OD). (3) Band pass filter: Homer2 provides fNIRS data filter because the useful blood oxygen information is generally about 0.1 Hz in the extremely low frequency, so the band pass is 0.01–0.2 Hz, so as to further remove the interference of respiratory and motion artifacts in fNIRS signal. (4) Optical density to blood oxygen concentration (DC): according to the Beer Lambert law, the optical density data are converted into hemoglobin concentration data.

To quantitatively compare the differences of cerebral blood oxygen response under different training conditions and to characterize the activation state of cerebral hemodynamics, the peak amplitude and integral area of two ROI were extracted for left- and right-hand motor imagery tasks before and after training. The general linear model (GLM) method was used to analyze the activation state of brain regions before and after training under the condition of left- and right-hand motor imagery task and draw the functional topological map based on the characteristics of cerebral blood oxygen.

## Results

### Clinical Scale Evaluation

All patients were assessed with clinical scales (from the attending doctors of Rehabilitation Department of Tianjin People's Hospital) in three standard assessment tasks. The results of basic scales are shown in Table 2.

**Table 2:** Changes of stroke patients with training duration were evaluated by the scale.

**ID**	**Group**	**Muscle strength assessment**			**Modified Barthel index**		
		**Day 1**	**Day 14**	**Day 28**	**Day 1**	**Day 14**	**Day 28**
S1	EG	2	2+	3	50	55	60
S2	EG	1–2	2	2+	30	30	45
S3	EG	2–	2	3	40	50	55
S4	EG	2–	2+	3	60	60	70
S5	CG	2	3	3	60	60	65
S6	CG	2–	2	2+	30	30	40
S7	CG	2	2+	2+	40	45	50

It can be seen from the evaluation results of the basic clinical scale in Table 2 that after 4 weeks of exercise rehabilitation training, the muscle strength assessment of all patients with stroke increased with the time of exercise rehabilitation training, and the living activity ability was also strengthened, but they did not reach the level of mild functional defect. It is speculated that it is related to the individual differences of patients and the duration of training. Specifically, after 4 weeks of NFT, the muscle strength assessment of patients in experimental group can be improved by more than 1 level, and the ability of living activities can be improved by more than 10 points. The muscle strength assessment of patients with stroke in the control group can also be improved by about 1 grade, and the ability of life activity can be improved by about 10 points. The results evaluated the long-term effect of NFT in stroke rehabilitation from the perspective of clinical motor behavior. Combining it with conventional clinical stroke rehabilitation training method is more effective than simple conventional training, but the advantage is not very significant. It is speculated that it is related to the individual differences of patients and the length of training.

### Data Processing and Analysis of EEG

**Figure 5:**
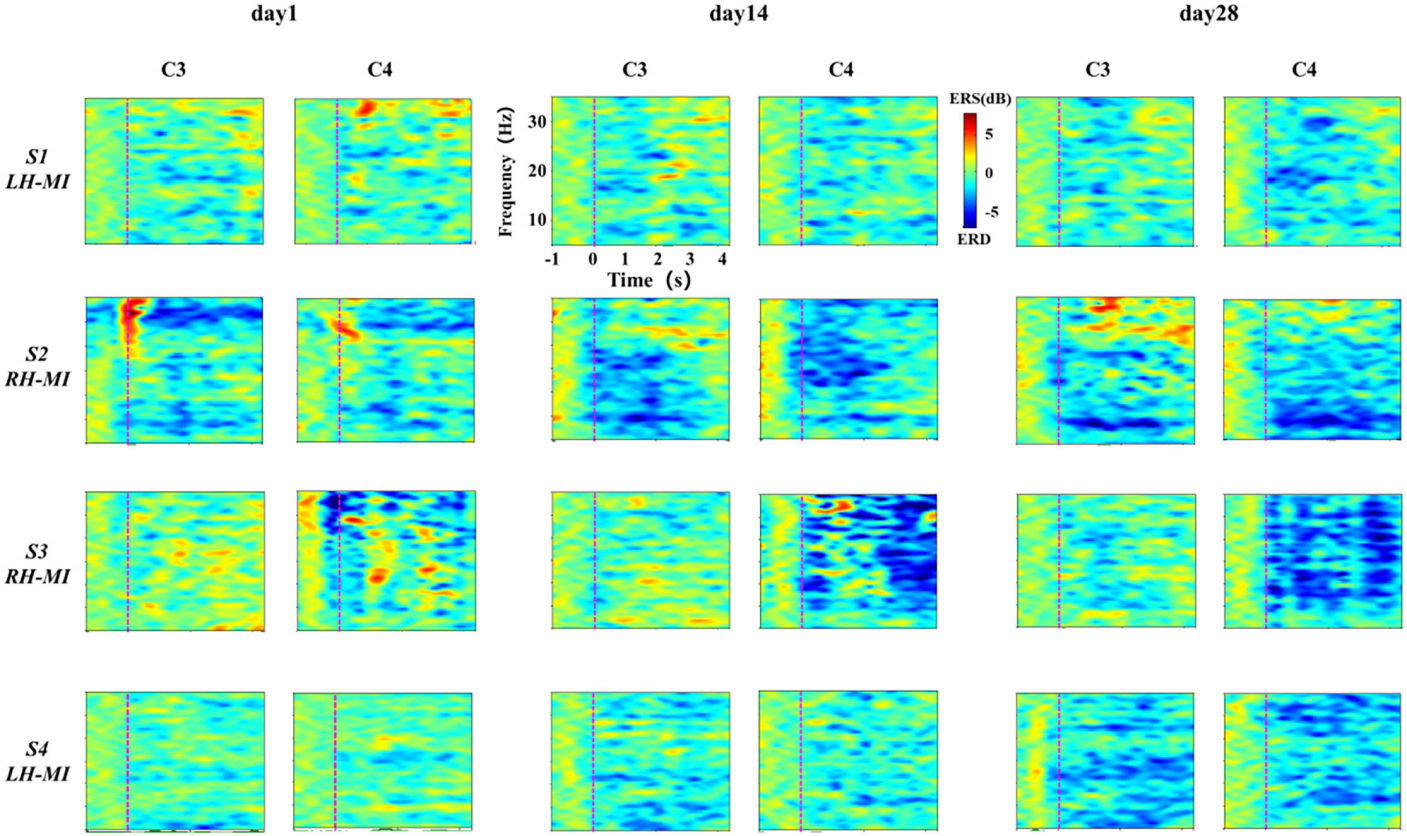
Results of EEG time-frequency response under standard assessment session in experimental group during long-term rehabilitation training.

**Figure 6:**
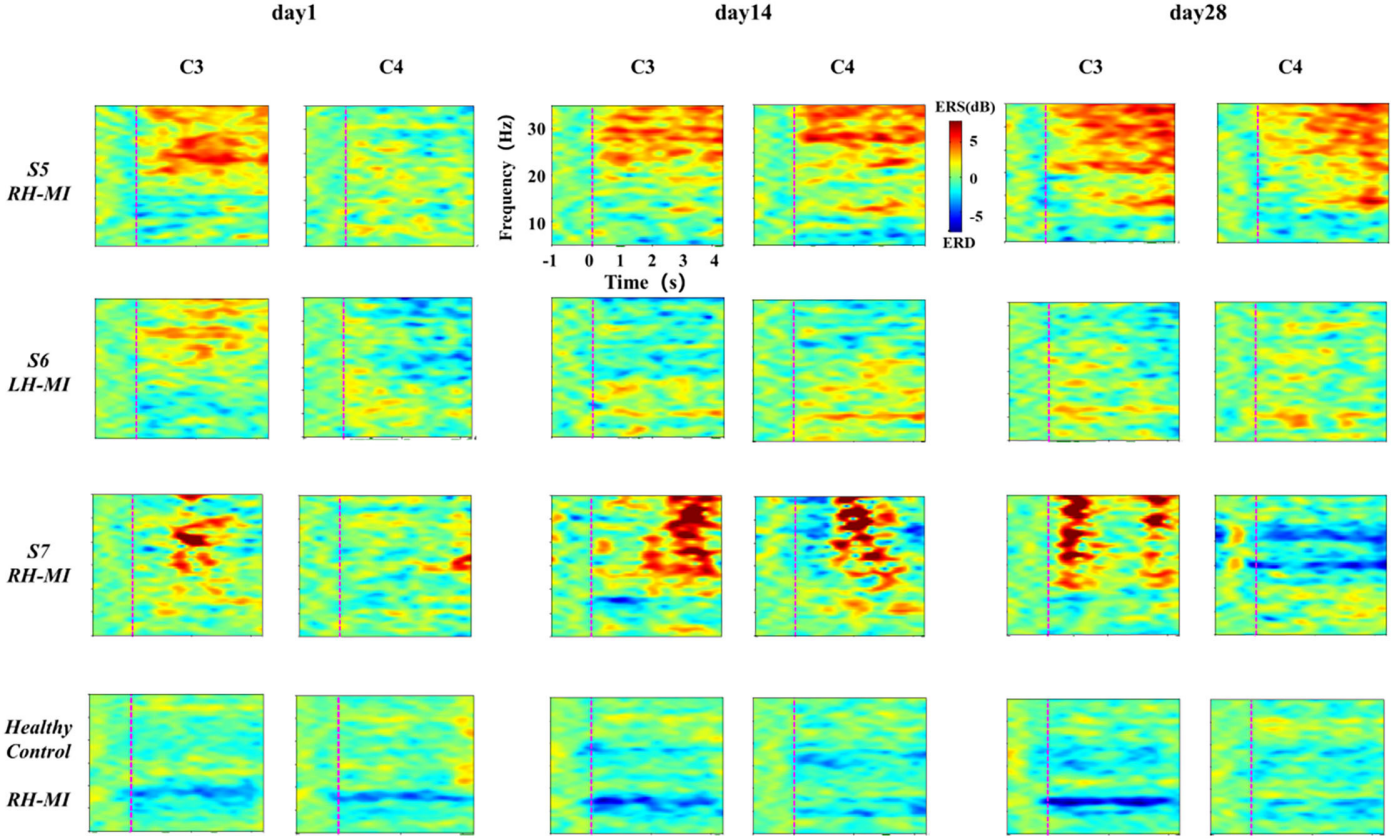
The results of EEG time-frequency response under standard assessment session in control group during long-term rehabilitation training.

In standard assessment session, we collected EEG signals of all subjects to characterize the change of the characteristic level induced by MI with the process of training. First, the task time node division was evaluated according to the standard, and the ERSP time-frequency diagrams of typical motor-related EEG channels (C3 and C4) under the basic MI of all subjects were calculated and drawn. Affected by great individual differences, the EEG time-frequency response results of all patients with stroke under the limb MI were given individually, while the healthy subjects showed their average results. The EEG time-frequency response results of patients in experimental group are shown in Figure 5, and the results of the control group are shown in Figure 6.

It can be seen from Figures 5, 6 that after 4 weeks of training, the time-frequency response induced by MI in the standard assessment session changed significantly. In the experimental group, the ERD characteristic pattern induced by MI was significantly enhanced along with the process of MI, but the regularity of enhancement was different, and the distribution frequency band was scattered. Specifically, the ERD characteristic pattern of patients S2 and S3 showed bilateral synchronous activation or even ipsilateral dominance in channels C3 and C4, which was further enhanced with the process of exercise rehabilitation training. The ERD characteristic pattern of patients S1 and S4 showed bilateral synchronous activation in channels C3 and C4, which was gradually enhanced with the process of exercise rehabilitation training. Finally, it can be shown that the contralateral dominance is obvious. In contrast, the ERD characteristic pattern induced by MI in the control group was weak, and there was obvious ERS. With the process of conventional motor rehabilitation training, the two characteristic patterns changed, and the distribution frequency band was also scattered. Specifically, patients S5 induced more obvious ERS characteristics in the process of MI, while the ERD characteristics were weak, which were further enhanced with the training process. Patients S6 and S7 showed some ERD characteristics, which were slightly enhanced with the training process. The healthy control group showed obvious ERD characteristic pattern with concentrated frequency distribution. With the process of NFT, this characteristic pattern was significantly enhanced and showed obvious contralateral dominance.

**Figure 7:**
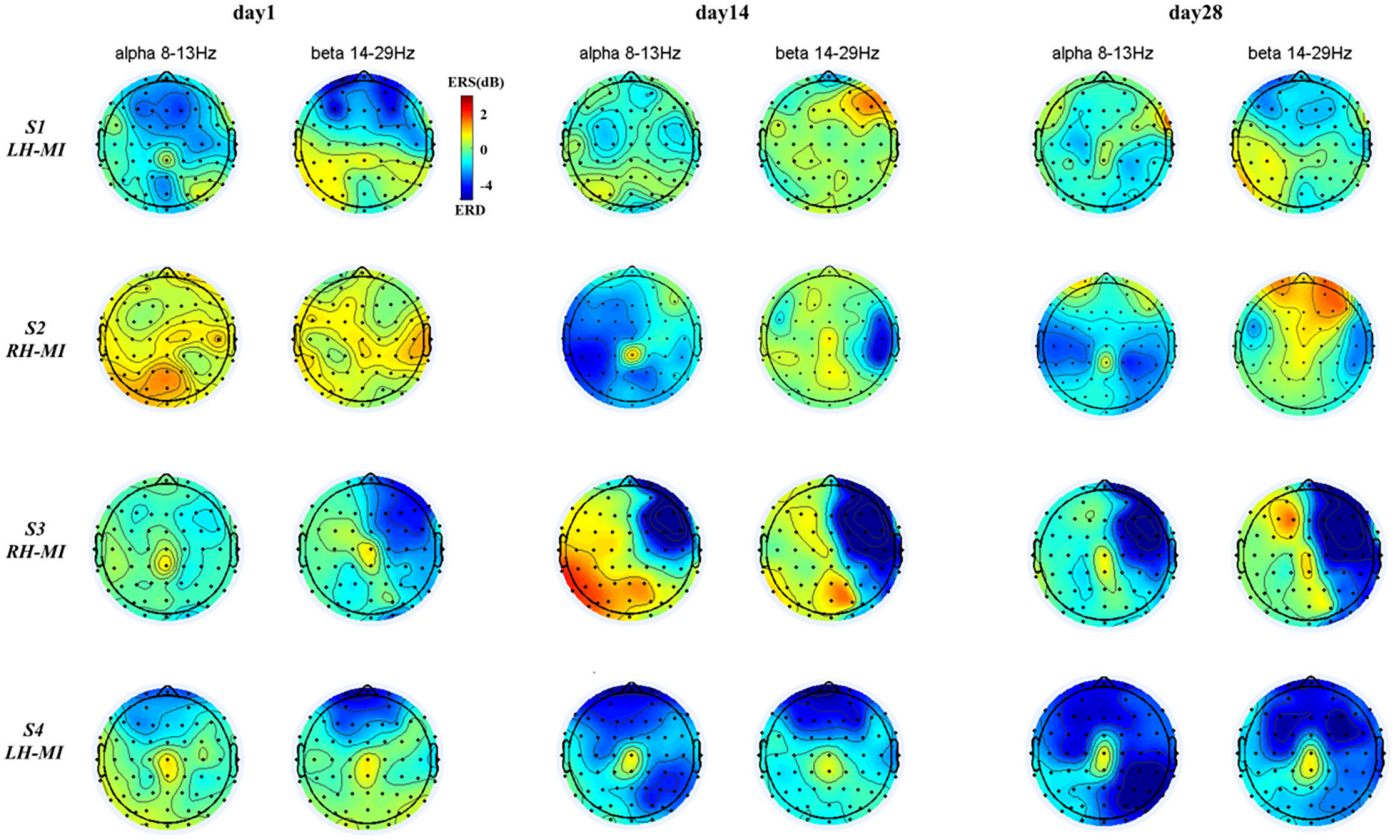
EEG brain mapping results of the experimental group under standard assessment session in long-term rehabilitation training.

**Figure 8:**
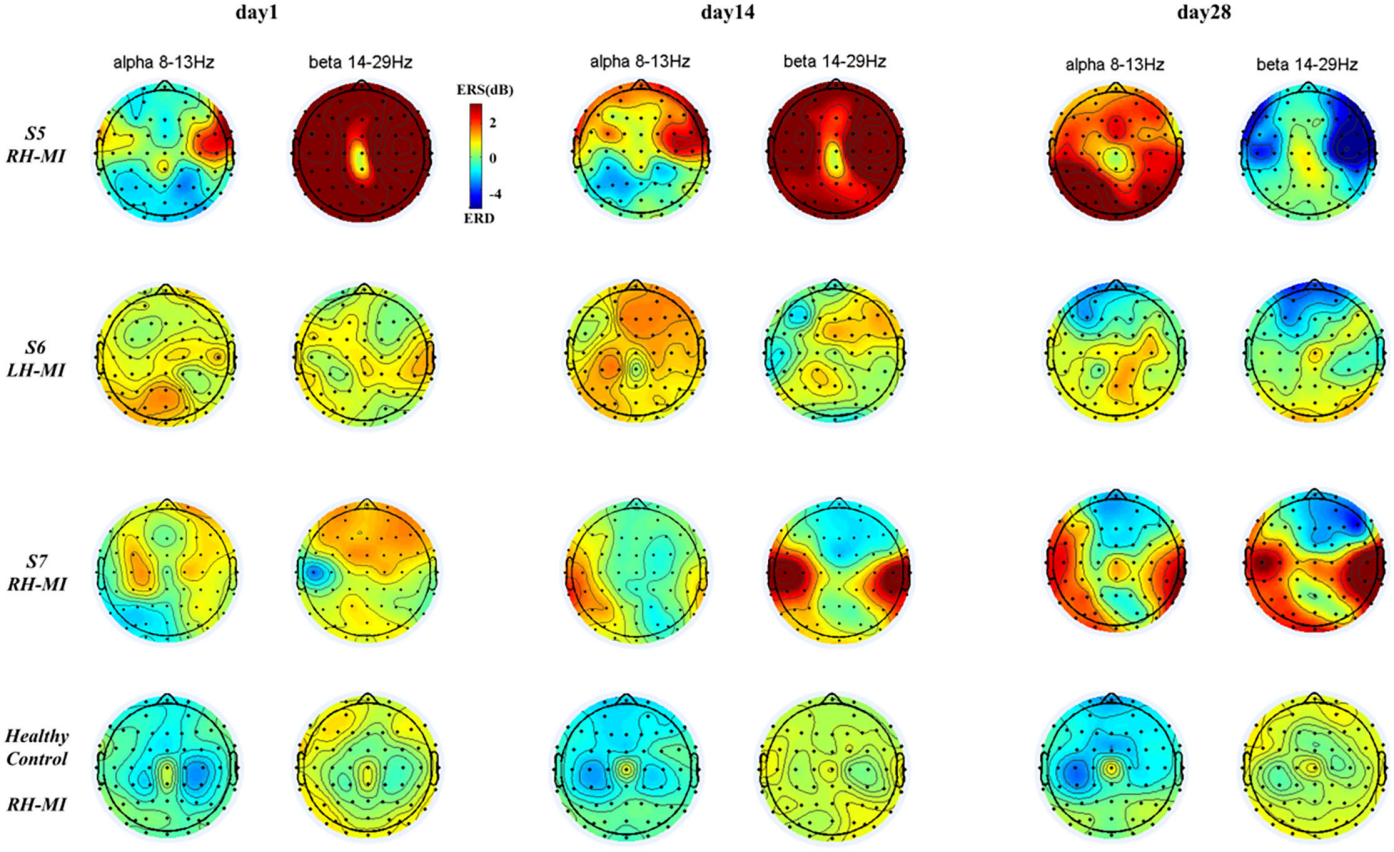
The results of EEG brain topographic map under standard assessment session in long-term rehabilitation training.

To further characterize the change of brain topological power response of all subjects during training, based on the time-frequency characteristic power of all EEG channels, the ERSP power of all subjects in typical frequency bands (alpha: 8–13 Hz, beta: 14–29 Hz) (Pfurtscheller and Da Silva, 1999; Graimann et al., 2002) was extracted, and the brain topographic map was drawn. The time-frequency power distribution of EEG brain topography in experimental group is shown in Figure 7, and the time-frequency power distribution of EEG brain topography in the control group and healthy control group is shown in Figure 8.

It can be seen from Figures 7, 8, after 4 weeks of training, all subjects had obvious changes in the distribution of brain topological power induced by MI in the standard assessment session. By comparing the topological power distribution of the whole brain, it is found that the ERD characteristic pattern of S1–S4 sensory motor-related areas in experimental group is significantly enhanced with the process of rehabilitation training, but the regularity of enhancement is different, and the power distribution area is relatively scattered. Specifically, the ERD characteristic pattern of S3, especially the alpha band power, showed ipsilateral activation in the whole brain, and this phenomenon was further enhanced with the process of rehabilitation training, while the ERD characteristic pattern of S1, S2, and S4 showed bilateral synchronous activation or contralateral dominance in the whole brain and gradually enhanced with the process of rehabilitation training. In contrast, the ERD characteristic pattern induced by MI in the control group was weak, and there was obvious ERS. With the process of conventional rehabilitation training, the two characteristic patterns changed, and the distribution area was also scattered. Specifically, in the whole brain of patients S5, the alpha band ERD characteristic pattern showed bilateral activation or contralateral dominance in day 1 and 14, but there was no regular enhancement trend. Patients S6 and S7 showed the coexistence of ERS and ERD, and the ERD/ERS in some areas (forehead and temporal lobe) increased with the training process. For the healthy control group, the ERD characteristic pattern is obvious, and the distribution area is relatively concentrated. With the NFT process, the alpha and beta band ERD characteristic patterns are enhanced and show obvious contralateral dominance.

**Figure 9:**
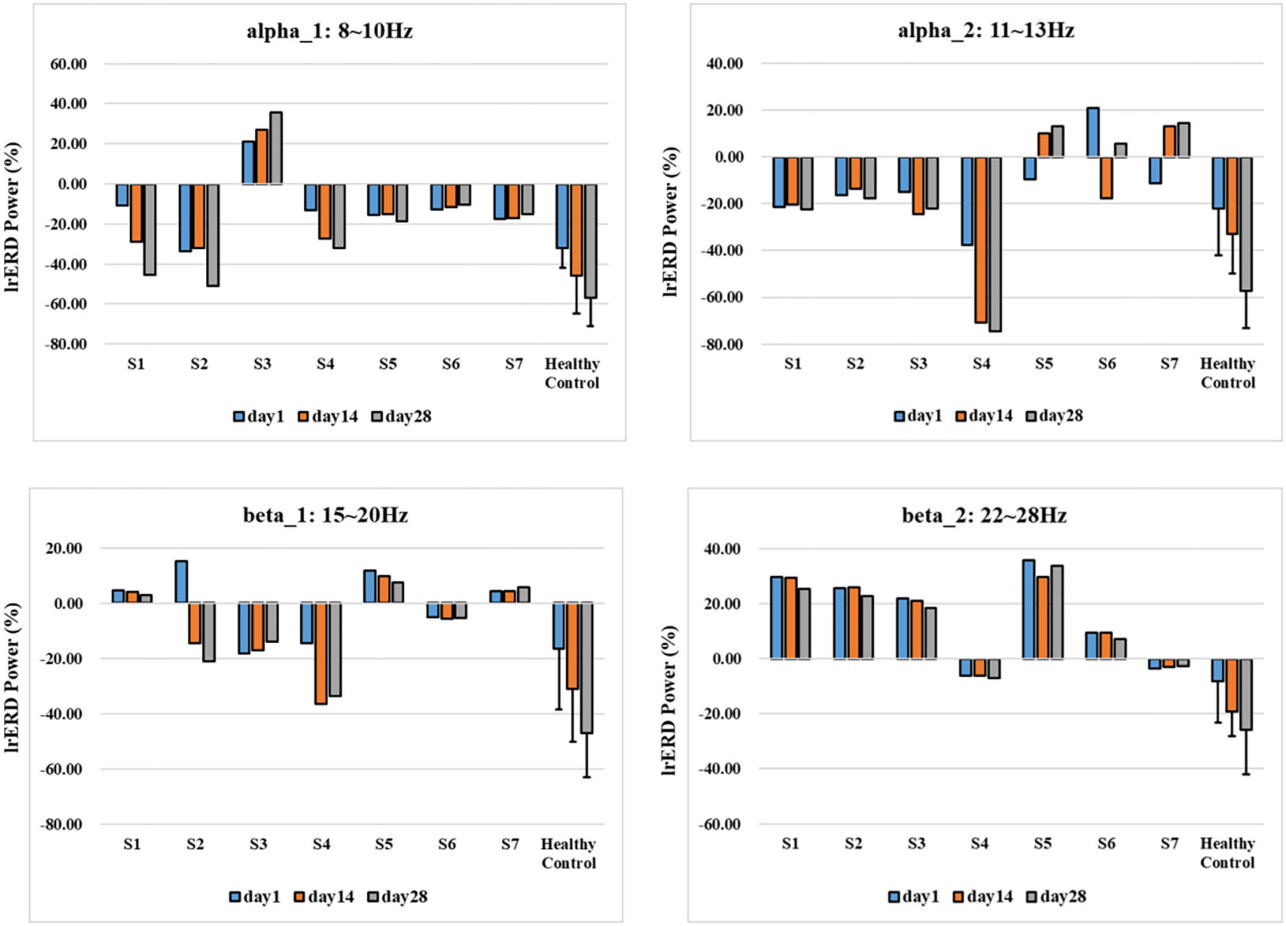
lrERD characteristic pattern results of standard assessment session in long-term training.

To quantify the activation effect of brain motor function, we further extracted lrERD characteristics of typical frequency bands of all patients and took the motor function-related channels C3 and C4 in the typical frequency band (alpha)_ 1:8–10 Hz, alpha_ 2:11–13 Hz, beta_ 1:15–20 Hz, beta_ 2. The difference of lrERD characteristics was calculated according to the imagination of the corresponding limb of the subjects, and then the differences of MI characteristic response modes of all subjects under the standard assessment session were compared. The results are shown in Figure 9.

As shown in Figure 9, corresponding to the results of time-frequency response and brain topological power distribution, the lrERD characteristic patterns of all subjects have changed to some extent with the process of training, and there are great individual differences in different typical frequency bands. Specifically, the lrERD of patients S1–S4 in the experimental group changed consistently in alpha1, and the characteristic mode level increased with the training process. Among them, patient S3 showed an obvious positive value, which was consistent with the ipsilateral activation enhancement phenomenon, indicating the lrERD characteristic activation level increased continuously with the training process. In contrast, the lrERD characteristic pattern of patients in the control group did not show the above consistent trend, and most of them showed the coexistence of positive and negative values. After long-term NFT, the characteristic pattern of lrERD was significantly enhanced in the healthy control group.

### Analysis of Cerebral Blood Oxygen Response Based on fNIRS

**Figure 10:**
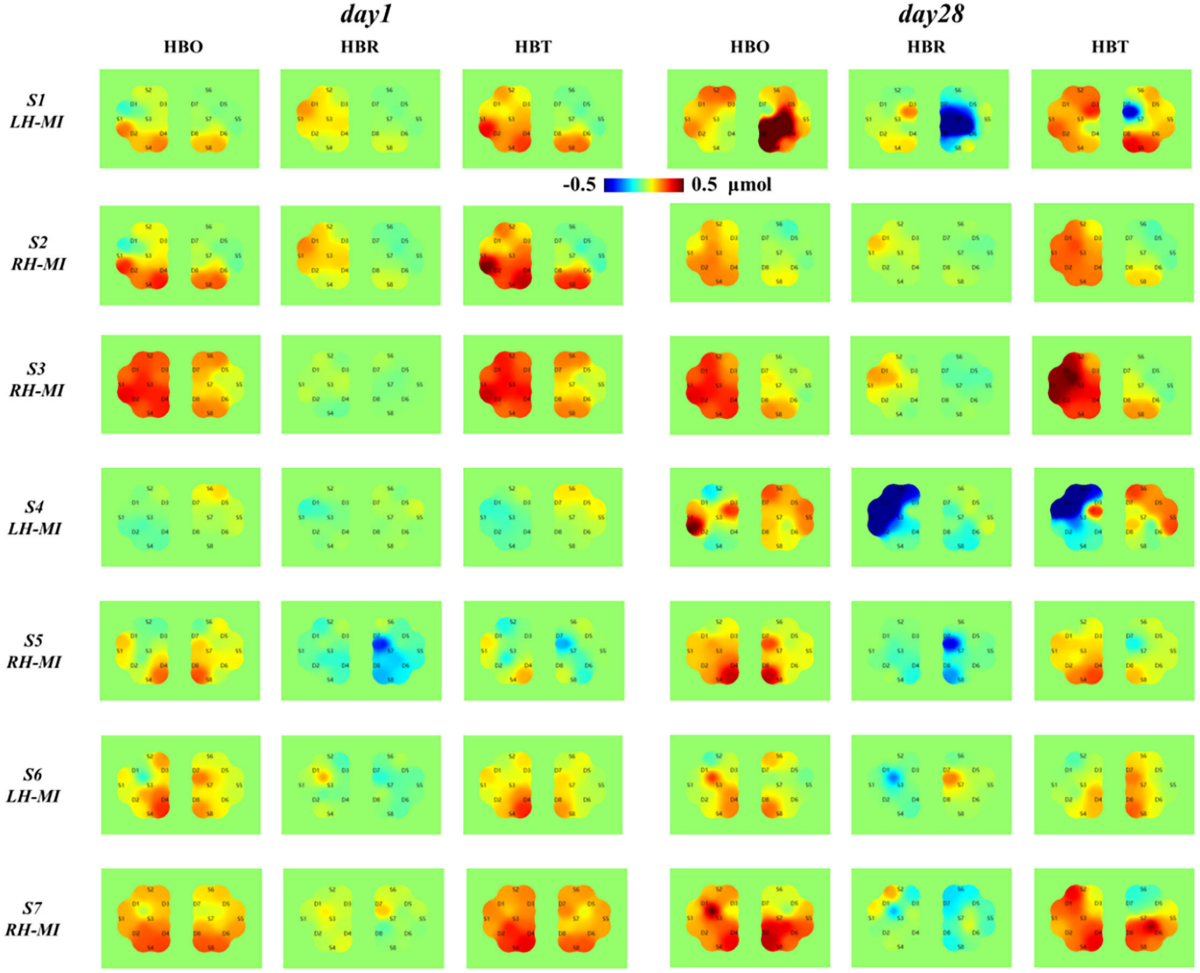
Topological distribution of cerebral blood oxygen concentration in patients with fNIRS before and after long-term exercise training.

To further characterize the changes of cerebral nerve function, the near-infrared cerebral blood oxygen information of all patients under day 1 and 28 standard assessment session was analyzed. Regions of interest (i.e., ROI1 and ROI2) were divided according to the corresponding limb movement task, and the topological structure of cerebral blood oxygen concentration changes of all patients based on fNIRS was calculated and drawn, as shown in Figure 10.

According to the topological analysis results of cerebral blood oxygen response in Figure 10, after 4 weeks of training, the characteristic response level of cerebral blood oxygen induced by MI of all patients has changed significantly, and the individual differences are large, and the change regularity is not completely consistent with the EEG time-frequency power response results. Specifically, the topological distribution of cerebral blood oxygen concentration from S1 to S4 in the experimental group showed obvious enhancement, and showed contralateral advantage. The cerebral blood oxygen level and contralateral advantage of patients in S1 and S4 increased significantly, which was consistent with the EEG time-frequency response results. The topological distribution of cerebral blood oxygen concentration from S5 to S7 in the control group also showed a certain enhancement, but it did not reach the level of significant improvement in the experimental group, and S5 and S6 showed a certain contralateral advantage, while S7 did not show a significant improvement and contralateral advantage. The results show that the introduction of multimodal NFT can significantly improve the cerebral blood oxygen level of patients and strengthen the induction of neural function rehabilitation.

## Discussion

Some studies have shown that the improvements experienced by the BCI treatment are due to neuroplastic changes in the central nervous system caused by closed-loop learning (Wolpaw, 2007; Biasiucci et al., 2018; Cervera et al., 2018). Closed-loop neurofeedback path refers to the establishment of information feedback path between the command output end and the user (Ang et al., 2015). The multimodal NFT method proposed in this study constructs a closed-loop neurofeedback path. In penalty stage, electrical stimulation is applied according to the real-time lrERD to promote the subjects to continuously improve the way of MI. In the reward stage, FES is applied according to the recognition results of online model based on CSP + SVM to promote the remodeling of neurons.

Under conventional clinical scale evaluation, Villafañe et al. (2018) verified the effectiveness of robot-assisted mobility in treating pain and spasticity in hand parallelism after stroke from the Barthel index, motility index, and other clinical scales. Biasiucci et al. used BCI-FES mode to help upper limb motor function recovery training of patients with stroke. After 6–12 months of follow-up study, the effectiveness of BCI-FES feedback training method in improving motor function and inducing neural plasticity rehabilitation was verified from the perspective of Fugl-Meyer and other clinical scales and brain electrophysiological functional network (Biasiucci et al., 2018). We recorded the patients' muscle strength assessment and modified Barthel index. The results showed that although there were large individual differences, the overall muscle strength level and living ability level were improved to a certain extent.

For the analysis of brain electrophysiological data, previous studies have shown that patients with stroke can elicit ERD/ERS during imagining the affected limb movement (Pfurtscheller and Aranibar, 1979; Neuper et al., 2006). Benzy et al. (2020) analyzed ERD/ERS and took phase locking value (PLV) as a feature to decode the direction of MI of the affected hand of patients with stroke. In this article, ERD and ERS are used to characterize the change of feature level induced by MI with the process of training. Meanwhile, lrERD is used as the feature parameter of the NFT penalty stage, and the activation effect of brain motor function is quantified. The results showed that there are obvious individual differences and abnormal phenomena, such as the whole brain strong ERS phenomenon in the beta band of S5 in Figure 8, which may be due to the high-frequency abnormal discharge of neurons caused by the disorder of synaptic transmission in patients with stroke. But overall, the ERD characteristic patterns of patients with C3 and C4 channels were enhanced in varying degrees, and the experimental group was better than the control group. For healthy subjects, the characteristic pattern of ERD was obvious. With the process of NFT, the characteristic pattern was significantly enhanced, and the contralateral advantage was obvious.

For cerebral blood oxygen response based on fNIRS analysis, previous studies collected EEG and fNIRS at the same time for joint analysis of brain electrophysiology and cerebral blood oxygen information. The results showed that after MI training, the activation degree of motor cortex and the connection level of related cortical functional network in patients with stroke were significantly improved (Kaiser et al., 2014; Delorme et al., 2019; Wang et al., 2019). In this article, multisensory NFT-MI training was carried out for patients with stroke. The results showed that the topological distribution of cerebral blood oxygen concentration in patients after training is significantly enhanced, and the contralateral advantage is more obvious.

As aforesaid, our goal was to verify the feasibility of long-term NFT system in assisting the rehabilitation of patients with stroke. We analyzed it from three aspects, namely, clinical scale, brain electrophysiology, and cerebral blood oxygen level. The results show that both the multimodal motor NFT method proposed in this study and the routine clinical rehabilitation training can induce the changes of cerebral nerve function, but there are differences in the specific change rules, that is, the former can induce the training from the motor center level, which can synchronously induce the changes of cortex-muscle function, making it close to the activation mode of motor conduction pathway in normal people, while the latter emphasizes the improvement of simple motor function and failure to consider the induction of synchronous functional rehabilitation of the central nervous system. However, there are two main limitations of this study. On the one hand, there are obvious individual differences in brain activity and change law, especially for patients with stroke. Because the focus, course of disease and other factors will affect the changes of nerve activation state and functional reorganization in the process of training, resulting in the activation of bilateral, ipsilateral, or contralateral brain areas in patients with stroke, and gradually form the corresponding steady-state mode. On the other hand, due to the insufficient number of patients recruited, we failed to draw statistically significant conclusions. We can only subjectively analyze the effects of NFT on brain electrophysiology and cerebral blood oxygen level. In view of the above limitations, in future research, we will expand the patient data to conduct more detailed data analysis and give a more scientific explanation.

## Data Availability Statement

The original contributions presented in the study are included in the article/supplementary files, further inquiries can be directed to the corresponding authors.

## Ethics Statement

The studies involving human participants were reviewed and approved by Tianjin People's Hospital. The patients/participants provided their written informed consent to participate in this study.

## Author Contributions

ZW, LC, BG, and DM were involved in the conception and design of the study, data interpretation, and critically reviewed the manuscript. ZW, SL, MX, and CC participated in the experimental data collection of this manuscript. CC and ZW were involved in the manuscript drafting and revision. ZW was involved in the data analysis for this manuscript. All authors contributed to the article and approved the submitted version.

## Funding

This study was supported by National Natural Science Foundation of China (Nos. 62006171, 82001939, and 81925020) and Natural Science Foundation of Tianjin (No. 20JCYBJC00930).

## Conflict of Interest

The authors declare that the research was conducted in the absence of any commercial or financial relationships that could be construed as a potential conflict of interest.

## Publisher's Note

All claims expressed in this article are solely those of the authors and do not necessarily represent those of their affiliated organizations, or those of the publisher, the editors and the reviewers. Any product that may be evaluated in this article, or claim that may be made by its manufacturer, is not guaranteed or endorsed by the publisher.
